# Transcriptomic analysis of tobacco-flavored E-cigarette and menthol-flavored E-cigarette exposure in the human middle ear

**DOI:** 10.1038/s41598-020-77816-2

**Published:** 2020-11-27

**Authors:** Jae-Jun Song, Yoon Young Go, Jong Kyou Lee, Bum Sang Lee, Su-Kyoung Park, Harry Jung, Jun Ho Lee, Jiwon Chang

**Affiliations:** 1 Department of Otolaryngology-Head and Neck Surgery, Korea University College of Medicine, Seoul, Korea; 2 Department of Otolaryngology-Head and Neck Surgery, Kangnam Sacred Heart Hospital, Hallym University College of Medicine, 948-1, Daerim 1-dong, Yeongdeunpo-gu, Seoul, 150-950 Korea; 3 Institute of New Frontier Research Team, Hallym Clinical and Translation Science Institute, Hallym University, Chuncheon, Republic of Korea

**Keywords:** Diagnostic markers, Genetics research, Translational research, Risk factors

## Abstract

Electronic cigarettes (e-cigarettes) are the most widely used electronic nicotine delivery systems and are designed to imitate smoking and aid in smoking cessation. Although the number of e-cigarette users is increasing rapidly, especially among young adults and adolescents, the potential health impacts and biologic effects of e-cigarettes still need to be elucidated. Our previous study demonstrated the cytotoxic effects of electronic liquids (e-liquids) in a human middle ear epithelial cell (HMEEC-1) line, which were affected by the manufacturer and flavoring agents regardless of the presence of nicotine. In this study, we aimed to evaluate the gene expression profile and identify potential molecular modulator genes and pathways in HMEEC-1 exposed to two different e-liquids (tobacco- and menthol-flavored). HMEEC-1 was exposed to e-liquids, and RNA sequencing, functional analysis, and pathway analysis were conducted to identify the resultant transcriptomic changes. A total of 843 genes were differentially expressed following exposure to the tobacco-flavored e-liquid, among which 262 genes were upregulated and 581 were downregulated. Upon exposure to the menthol-flavored e-liquid, a total of 589 genes were differentially expressed, among which 228 genes were upregulated and 361 were downregulated. Among the signaling pathways associated with the differentially expressed genes mediated by tobacco-flavored e-liquid exposure, several key molecular genes were identified, including IL6 (interleukin 6), PTGS2 (prostaglandin-endoperoxide synthase 2), CXCL8 (C-X-C motif chemokine ligand 8), JUN (Jun proto-oncogene), FOS (Fos proto-oncogene), and TP53 (tumor protein 53). Under menthol-flavored e-liquid treatment, MMP9 (matrix metallopeptidase 9), PTGS2 (prostaglandin-endoperoxide synthase 2), MYC (MYC proto-oncogene, bHLH transcription factor), HMOX1 (heme oxygenase 1), NOS3 (nitric oxide synthase 3), and CAV1 (caveolin 1) were predicted as key genes. In addition, we identified related cellular processes, including inflammatory responses, oxidative stress and carcinogenesis, under exposure to tobacco- and menthol-flavored e-liquids. We identified differentially expressed genes and related cellular processes and gene signaling pathways after e-cigarette exposure in human middle ear cells. These findings may provide useful evidence for understanding the effect of e-cigarette exposure.

## Introduction

Electronic cigarettes (e-cigarettes) are the most widely used electronic nicotine delivery system (ENDS) and were designed to imitate and reduce conventional smoking. An e-cigarette is composed of a battery, a vaporizing chamber and an electronic liquid (e-liquid). When e-cigarettes heat e-liquids, aerosols are delivered to users when they inhale. The main components of the e-liquid are propylene glycol, vegetable glycerin, and flavoring agents with or without the addition of nicotine. These devices entered the market in 2007^1,2^, but the market has been growing rapidly, and ENDS use has been reported to have more than doubled among young adults^3^.

Although these devices were designed to reduce smoking, there is increasing evidence that nicotine exposure delivered by e-cigarettes can induce addiction and activate multiple biological pathways in a manner similar to conventional smoking^3^. Heating the e-liquid produces toxic, oxidative, inflammatory aerosols^4^, and e-cigarettes can expose users to chemicals including volatile compounds, carbonyl compounds, formaldehyde, acrolein and heavy metals, which are known to have adverse health effects^5–7^. Recent studies regarding the health effects of e-cigarettes have reported asthma exacerbation and increases in chronic bronchitis^8–10^, and an outbreak of e-cigarette product use-associated lung injury (EVALI) occurred in mid-2019. Patients with EVALI present with respiratory, gastrointestinal and constitutional symptoms^11^. Most of the studies regarding the health effects of e-cigarettes do not provide evidence of long-term effects, but in the short term, e-cigarette exposure has been shown to increase the odds ratio of myocardial infarction^12^ and to be associated with stroke^13^. Additionally, there is growing evidence that e-cigarette aerosols present a carcinogenic potential and deregulate cancer-associated genes^13^.

Propylene glycol and glycerin are humectants from which pulmonary irritants and carcinogenic carbonyl compounds are produced when heated. Heavy metals may be leached from the metals contained in the heating coils and cartridge casings of e-cigarettes^11^. Flavoring agents are considered safe for ingestion, but there is a lack of safety data under inhalational exposure^11^. Studies reporting the cytotoxicity of e-liquids to human pulmonary fibroblasts, human embryonic stem cells and mouse neural stem cells have indicated that their cytotoxicity is related to the flavor chemicals^14,15^. Others have reported that the flavoring chemicals in e-liquid induce transcriptomic changes and perturb cilium function in the primary normal human bronchial epithelium (NHBE)^16^. Studies on e-cigarette aerosols conducted in human bronchial epithelial (HBE) cells have reported discrete transcriptomic signatures in the presence or absence of added nicotine^17^ and have indicated that alterations in cellular glycerophospholipid biosynthesis are an important consequence of aerosol exposure.

The effect of e-cigarettes on the upper airway or middle ear is considerably underestimated. The middle ear is connected to the nasopharynx and nasal cavity through the Eustachian tube and is vulnerable to bacterial infection and environmental pollutants. As conventional smoking is a well-known cause of otitis media, the use of e-cigarettes may influence the middle ear mucosa and increase the occurrence of otitis media. In our previous study^6^, we evaluated diverse e-liquids available in the market and showed that e-liquids influenced and had adverse effects on a human middle ear epithelial cell (HMEEC-1) line. E-cigarettes with or without nicotine decreased cell viability and caused cytotoxicity, which was variable among different manufacturers, solvent proportions and flavoring agents. Additionally, among various flavored e-liquids, menthol-flavored e-liquids were the most cytotoxic and decreased cell viability more than the other flavors.

In this study, we evaluated the gene expression profile and identified potential molecular modulator genes and pathways in human middle ear epithelial cells (HMEEC-1) under exposure to e-liquids through mRNA sequencing analysis. We also compared the gene expression profiles of two different flavored e-liquids (tobacco-flavored and menthol-flavored e-liquids) to identify the difference in the gene expression profile.

## Results

**Figure 1:**
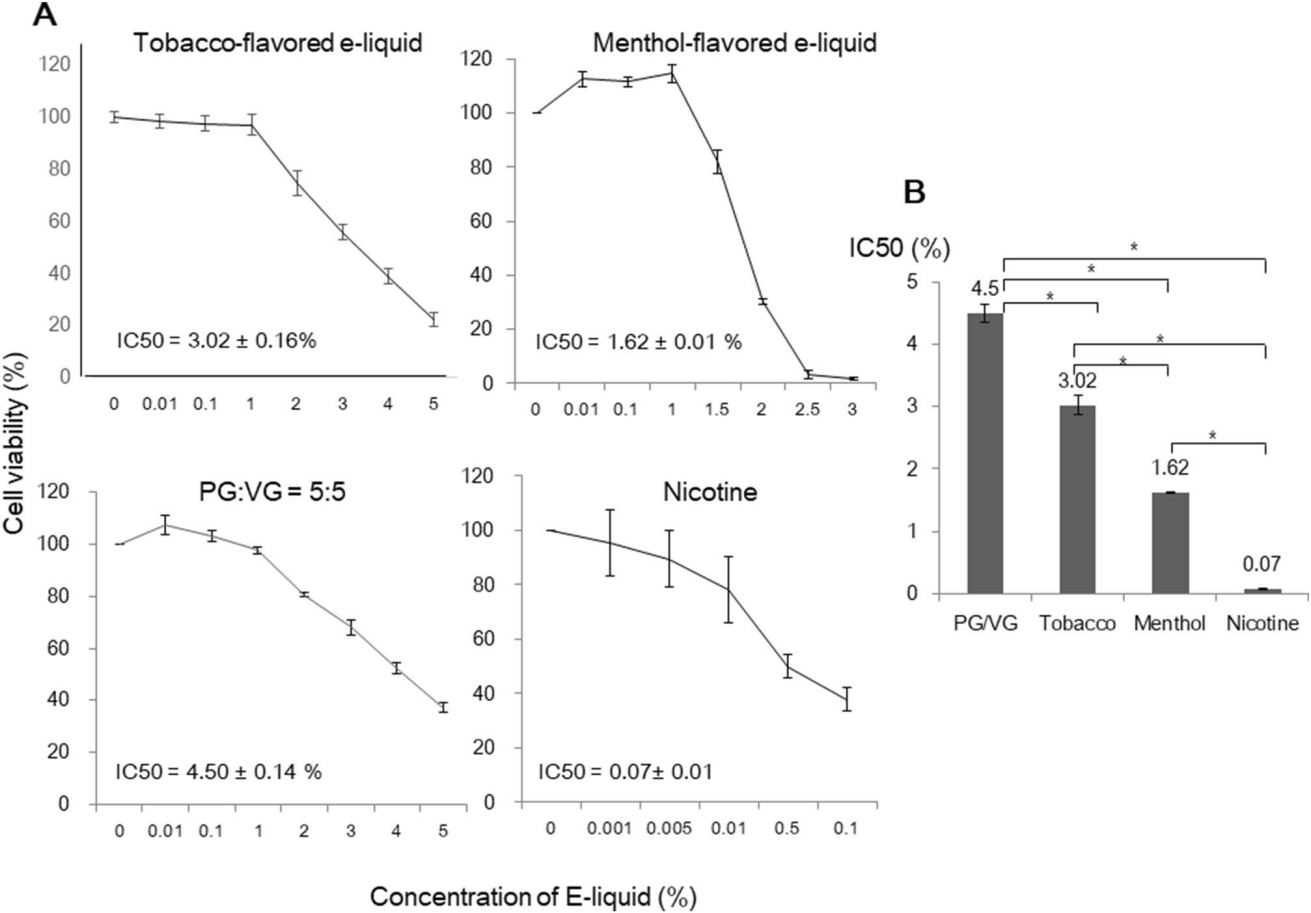
Cytotoxic effect of e-liquids on HMEEC-1 cells according to CCK-8 analysis. (**A**) We assessed cell viability following exposure to tobacco-flavored e-liquid, menthol-flavored e-liquid, solvent (PG:VG = 5:5), and nicotine at different concentrations. (**B**) The IC50 values for each type of solution were 3.02 ± 0.16%, 1.62 ± 0.01%, 4.50 ± 0.14% and 0.07 ± 0.01 mg/mL, respectively, and these concentrations were used for further analysis (*p* < 0.05).

**Figure 2:**
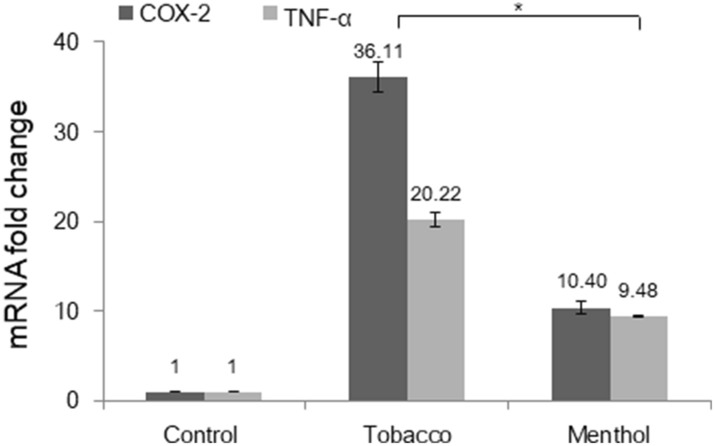
E-liquids stimulated the expression of inflammatory cytokines in HMEEC-1. Cells were treated with tobacco-flavored e-liquid and menthol-flavored e-liquid at the IC50 concentration for 24 h. Quantitative real-time PCR was performed to evaluate inflammatory cytokine gene expression levels. The expression levels of COX-2 and TNF-α increased when cells were exposed to the e-liquids, and the mRNA expression of cytokines was significantly elevated in the tobacco-flavored e-liquid group (*p* < 0.05).

The main components of e-liquids are propylene glycol (PG), vegetable glycerin (VG), and flavoring agents, with/without the addition of nicotine. In previous studies, when we analyzed the toxicity of the solvent alone (PG:VG = 5:5) versus tobacco-flavored e-liquids (without nicotine) and menthol-flavored e-liquid (without nicotine), the average IC50 values in the flavored e-liquids were decreased^5^, indicating that flavor-added e-liquids are toxic and that between these two flavors, menthol-flavored e-liquids are more cytotoxic. In the present study, the effect of the components of e-liquids on cell viability was measured again via CCK-8 analysis, and the IC50 values were obtained. The IC50 value of the solvent of the e-liquids (PG:VG = 5:5) was 4.50 ± 0.14%, and that of nicotine was 0.07 ± 0.01 mg/mL. Additionally, the IC50 values of tobacco-flavored e-liquid and menthol-flavored e-liquids were 3.02 ± 0.16% and 1.62 ± 0.01%, respectively (Fig. 1). These IC50 values were subjected to further analysis. The IC50 value of the menthol-flavored e-liquid was lower than that of the tobacco-flavored e-liquid. However, when we identified the expression levels of inflammatory cytokines in HMEEC-1 after e-liquid exposure, the expression levels of COX-2 and TNF-α were found to be higher in the tobacco-flavored e-liquid than in the menthol-flavored e-liquid, indicating that the mechanism underlying the observed cytotoxicity differs between the two flavors (Fig. 2).

### E-liquid-related gene expression profile in HMECC-1

**Figure 3:**
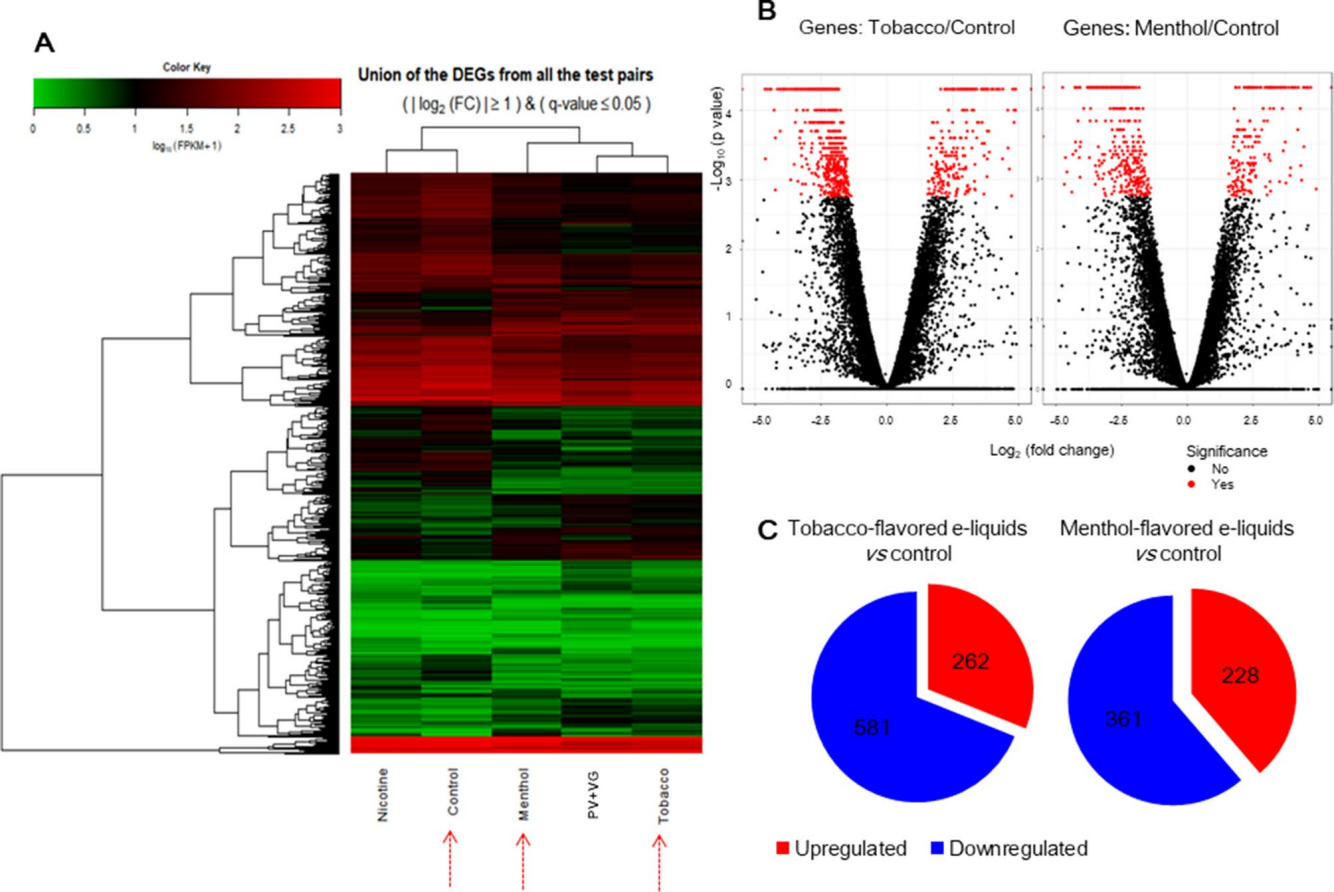
Heatmap and volcano plots of RNA sequencing results. (**A**) In the heatmap, the expression values of the selected DEGs (differentially expressed genes) in log_10_(FPKM) units were compared across samples (nicotine, solvent (PG + VG), menthol-flavored e-liquid, tobacco-flavored e-liquid and control). Red indicates transcripts with high expression, and green indicates transcripts with low expression. (**B**) Volcano plots of log_2_ (fold change) and −log_10_ (*p*-value) values are presented to show the changes in gene expression and their significance. (**C**) DEGs of tobacco-flavored and menthol-flavored e-liquids. Under tobacco-flavored e-liquid exposure, a total of 843 genes were differentially expressed with fold changes of | Log2 |> 1. Among these genes, 262 were upregulated, and 581 were downregulated. Menthol-flavored e-liquid exposure resulted in 228 upregulated genes and 361 downregulated.

To determine whether gene expression is altered in response to exposure to different flavored e-liquids, the solvent or nicotine, we investigated gene expression levels through mRNA sequencing analysis and generated a heat map using the average values of two replicates for all five samples (Fig. 3A). The heatmap of the expression values of the selected DEGs in log10 (FPKM) units was compared across genes and samples (fold change > 2.0 and Q < 0.05). The differentially expressed genes (DEGs) between the two selected biological conditions were analyzed (Control-VS-Menthol, Control-VS-Nicotine, Control-VS-PV, Control-VS-Tobacco, Menthol-VS-Nicotine, Menthol-VS-PV, Menthol-VS-Tobacco, Nicotine-VS-PV, Nicotine-VS-Tobacco, PV-VS-Tobacco). Since our study was aimed to identify the gene expression according to two different flavored e-liquids, volcano plots were drawn for comparisons across control and two different flavored e-liquids (Fig. 3B). Both tobacco-flavored and menthol-flavored e-liquids induced significant changes in gene expression. Under tobacco-flavored e-liquid exposure, a total of 843 genes were differentially expressed with fold changes of | Log2 |> 1 in response to e-liquid exposure (Q < 0.05) (Fig. 3C, Supplementary dataset S1). Among these genes, 262 were upregulated, and 581 were downregulated (Fig. 3C). Menthol-flavored e-liquid exposure resulted in 228 upregulated genes and 361 downregulated genes with fold changes of | Log2 |> 1 (Q < 0.05) (Fig. 3C, Supplementary dataset S2).

KEGG pathway analysis revealed that tobacco-flavored e-liquid exposure caused a prominent change in the expression of genes involved in translation, infection, cell cycle/apoptosis-related signal transduction, cancer and the regulation of metabolic pathways (Table 1). Menthol-flavored e-liquid exposure resulted in a prominent change in the expression of genes involved in metabolic pathways, cancer, inflammation, and focal adhesion (Table 2).

**Table 1:** Analysis of enrichment of KEGG pathways and involved genes against tobacco-flavored e-liquids.

Name	Involved genes	*p*-value
Ribosome	RPL18, RPSA, RPL13, RPL35, RPL27, RPS9, RPS2, RPS5, MRPS2, RPL29, MRPL24, MRPL23, MRPL12, MRPL28, RPL18A, RPL13A, RPLP0, RPL8, RPL10, RPS10, RPL7A	0.000
Glycolysis/Gluconeogenesis	PKM, GPI, LDHA, PFKL, GCK, ALDH1B1, PGM1, ALDH3B2, ALDH3B1, ENO1	0.003
Arginine and proline metabolism	PYCRL, CKMT1A, CKMT1B, SRM, ALDH1B1, GAMT, NOS3, PRODH, CKB	0.006
beta-Alanine metabolism	AOC2, GAD1, AOC3, SRM, ALDH1B1, ALDH3B2, ALDH3B1	0.006
Bladder cancer	CXCL8, HBEGF, CDKN2A, FGFR3, TP53, THBS1, CDK4, MYC	0.007
Phenylalanine metabolism	AOC2,AOC3, ALDH3B2, ALDH3B1, MIF	0.012
Pertussis	FOS, IL6, IL23A, C3, LY96, JUN, CXCL8, PYCARD, CALML5, CD14	0.022
Galactose metabolism	HKDC1, AKR1B10, B4GALT2, PFKL, GCK, PGM1	0.023
Legionellosis	IL6, CXCL3, CXCL2, C3, PYCARD, CXCL8, CD14, HSPA8	0.028
p53 signaling pathway	CCNG2, GADD45A, STEAP3, CDKN2A, TP53, DDB2, SFN, THBS1, CDK4	0.030
Starch and sucrose metabolism	GPI, GCK, PYGL, PGM1, PYGB	0.033
Salmonella infection	FOS, IL6, CXCL3, JUN, CXCL2, CXCL8, WASL, PYCARD, FLNB, CD14	0.039
Alanine, aspartate and glutamate metabolism	GFPT1, ASNS, GAD1, GLUL, ADSL, CAD	0.042
Small cell lung cancer	TRAF1, PTGS2, COL4A2, LAMA5, LAMC3, RXRA, TP53, CDK4, MYC, PIK3R2	0.044
DNA replication	MCM7, LIG1, POLD2, MCM2, MCM3, MCM5	0.046

**Table 2:** Analysis of enrichment of KEGG pathways and involved genes against menthol-flavored e-liquids.

Name	Involved genes	*p*-value
Arginine and proline metabolism	PYCRL, CKMT1A, ALDH7A1, CKMT1B, SRM, ALDH1B1, GAMT, NOS3, PRODH, CKB	0.000
Metabolic pathways	YP24A1, CKMT1B, PTGS2, CYC1, CAD, GPAT3, GLDC, CKB, AKR1C3, TRAK2, NOS3, PLCB2, IMPDH2, CYP1A1, PFKL, HKDC1, ALDH3B2, NME4, ALDH7A1, MGAT3, ALDH1B1, AKR1B10, ABAT, SLC27A5, PRODH, ALOX12, ME1, PYCRL, GCNT3, GCNT2, GCLC, AHCY, SORD, ASS1, SRM, GPAA1, UPP1, ASNS, ATP5G1, GCLM, PFAS, TYMP, DGKG, FASN, PNPO, HSD17B3, GALNT18, PNLIPRP3, B4GALNT1, HSD17B8, ST6GAL1, B4GALT2, APRT, CKMT1A, GCK, NDUFV1, GFPT1, POLD1, CSGALNACT2, PLCG2, POLD2, SMPD1, GAMT, CYP4F3, GK, RDH16, NNMT	0.000
Bladder cancer	TYMP, FGFR3, MMP9, HBEGF, THBS1, MYC, MMP1	0.003
Inflammatory mediator regulation of TRP channels	TRPV3, PLCG2, ASIC3, ASIC1, MAPK10, CALML5, PLCB2, ITPR1, PIK3R2, ALOX12	0.013
ECM-receptor interaction	COL4A2, LAMB3, LAMA5, LAMC3, TNC, HSPG2, AGRN, ITGA4, THBS1	0.007
Proteoglycans in cancer	CAV1, WNT10B, TFAP4, MMP9, HSPG2, FLNC, ITPR1, CTSL, SMO, ANK1, PLCG2, HBEGF, THBS1, MYC, PIK3R2	0.013
Glycerolipid metabolism	ALDH7A1, ALDH1B1, AKR1B10, DGKG, GK, GPAT3, PNLIPRP3	0.018
Steroid hormone biosynthesis	AKR1C3, AKR1C2, CYP1A1, HSD11B2, HSD17B3, AKR1C1, HSD17B8	0.018
Galactose metabolism	B4GALT2, PFKL, GCK, HKDC1, AKR1B10	0.022
beta-Alanine metabolism	ALDH7A1, SRM, ALDH1B1, ABAT, ALDH3B2	0.024
Mineral absorption	HMOX1, MT1B, MT1X, FTH1, FTL, MT1F	0.024
Small cell lung cancer	TRAF1, COL4A2, LAMB3, PTGS2, LAMA5, LAMC3, MYC, PIK3R2	0.034
Focal adhesion	COL4A2, CAV1, TNC, ITGA4, MAPK10, FLNC, LAMB3, LAMA5, CCND2, LAMC3, RAC3, THBS1, PARVB, PIK3R2	0.036
Alanine, aspartate and glutamate metabolism	ASS1, GFPT1, ABAT, CAD, ASNS	0.036

For the tobacco-flavored e-liquid group, the GO annotations of the predicted targets enriched among the 838 genes that were mappable to DAVID were selected according to a |fold-change|≥ 2 and Q-value ≤ 0.05 compared to the control. For the menthol-flavored e-liquid group, the GO annotations of the predicted targets enriched among the 586 genes were also selected according to the above criteria. The functional annotations were categorized into biological processes, cellular components and molecular functions, and only the top 10 GO terms showing the smallest *p*-values were considered. This analysis revealed similar patterns for the two flavored e-liquids and several enriched functional categories and target genes, including genes involved in binding, antioxidative activity, cell communication, and intracellular signal transduction (Tables 3, 4).

**Table 3:** Go annotation of predicted targets in tobacco-flavored e-liquid.

Term	Count	%	*p*-value
**Molecular function**			
GO:0003735 ~ structural constituent of ribosome	28	3.341	3.99E-06
GO:0016614 ~ oxidoreductase activity, acting on CH-OH group of donors	20	2.387	1.68E-05
GO:0005102 ~ receptor binding	102	12.172	2.01E-05
GO:0019838 ~ growth factor binding	19	2.267	2.29E-05
GO:0016616 ~ oxidoreductase activity, acting on the CH-OH group of donors, NAD or NADP as acceptor	18	2.148	2.50E-05
GO:0048037 ~ cofactor binding	29	3.461	6.38E-05
GO:0005198 ~ structural molecule activity	61	7.279	6.94E-05
GO:0050839 ~ cell adhesion molecule binding	40	4.773	1.91E-04
GO:0050840 ~ extracellular matrix binding	10	1.193	6.37E-04
GO:0072341 ~ modified amino acid binding	11	1.313	1.31E-03
**Biological processes**			
GO:0055114 ~ oxidationreduction process	89	10.621	1.78E-09
GO:1901566 ~ organonitrogen compound biosynthetic process	113	13.484	2.23E-09
GO:0006364 ~ rRNA processing	38	4.535	3.08E-09
GO:0016072 ~ rRNA metabolic process	38	4.535	6.25E-09
GO:0006082 ~ organic acid metabolic process	82	9.785	9.19E-09
GO:0019752 ~ carboxylic acid metabolic process	75	8.950	3.72E-08
GO:0042594 ~ response to starvation	26	3.103	3.78E-08
GO:0043436 ~ oxoacid metabolic process	75	8.950	4.80E-08
GO:0009991 ~ response to extracellular stimulus	48	5.728	6.31E-08
GO:0006793 ~ phosphorus metabolic process	202	24.105	1.03E-07
**Cellular component**			
GO:1903561 ~ extracellular vesicle	202	24.105	4.68E-09
GO:0043230 ~ extracellular organelle	202	24.105	4.76E-09
GO:0044421 ~ extracellular region part	258	30.788	1.18E-08
GO:0070062 ~ extracellular exosome	199	23.747	1.45E-08
GO:0031988 ~ membranebounded vesicle	238	28.401	1.58E-07
GO:0005829 ~ cytoso	214	25.537	2.67E-05
GO:0005576 ~ extracellular region	278	33.174	2.82E-05
GO:0031012 ~ extracellular matrix	48	5.728	6.23E-05
GO:0031967 ~ organelle envelope	85	10.143	1.30E-04
GO:0031975 ~ envelope	85	10.143	1.52E-04

The top 10 most enriched GO terms are listed in terms for biological process, cellular component, and molecular function based on *p*-values.

**Table 4:** Go annotation of predicted targets in menthol-flavored e-liquid.

Term	Count	%	*p*-value
**Molecular function**			
GO:0016614 ~ oxidoreductase activity, acting on CH-OH group of donors	15	2.560	1.32E-04
GO:0016616 ~ oxidoreductase activity, acting on the CH-OH group of donors, NAD or NADP as acceptor	13	2.218	3.79E-04
GO:0005102 ~ receptor binding	71	12.116	4.36E-04
GO:0005509 ~ calcium ion binding	40	6.826	9.26E-04
GO:0050662 ~ coenzyme binding	16	2.730	9.42E-04
GO:0048037 ~ cofactor binding	20	3.413	1.35E-03
GO:0033764 ~ steroid dehydrogenase activity, acting on the CH-OH group of donors, NAD or NADP as acceptor	6	1.024	1.50E-03
GO:0016709 ~ oxidoreductase activity, acting on paired donors, with incorporation or reduction of molecular oxygen, NAD(P)H as one donor, and incorporation of one atom of oxygen	7	1.195	2.36E-03
GO:0016229 ~ steroid dehydrogenase activity	6	1.024	2.85E-03
GO:0047086 ~ ketosteroid monooxygenase activity	3	0.512	3.02E-03
**Biological processes**			
GO:0010033 ~ response to organic substance	153	26.109	9.81E-12
GO:0070887 ~ cellular response to chemical stimulus	145	24.744	2.20E-11
GO:0009966 ~ regulation of signal transduction	144	24.573	3.80E-10
GO:0010646 ~ regulation of cell communication	155	26.451	4.54E-10
GO:0023051 ~ regulation of signaling	156	26.621	7.93E-10
GO:0071310 ~ cellular response to organic substance	118	20.137	8.13E-09
GO:1901700 ~ response to oxygen-containing compound	88	15.017	1.07E-08
GO:0023057 ~ negative regulation of signaling	73	12.457	1.00E-07
GO:0010647 ~ positive regulation of cell communication	88	15.017	1.55E-07
GO:0009968 ~ negative regulation of signal transduction	68	11.604	1.77E-07
**Cellular component**			
GO:0044421 ~ extracellular region part	196	33.447	2.19.E-10
GO:0005576 ~ extracellular region	214	36.519	5.55.E-08
GO:1903561 ~ extracellular vesicle	142	24.232	4.58.E-07
GO:0043230 ~ extracellular organelle	142	24.232	4.63.E-07
GO:0070062 ~ extracellular exosome	141	24.061	5.74.E-07
GO:0031988 ~ membrane bounded vesicle	167	28.498	5.72.E-06
GO:0005578 ~ proteinaceous extracellular matrix	28	4.778	1.11.E-04
GO:0005615 ~ extracellular space	74	12.628	3.42.E-04
GO:0005829 ~ cytosol	148	25.256	5.52.E-04
GO:0031012 ~ extracellular matrix	33	5.631	1.24.E-03

The top 10 most enriched GO terms are listed in terms for biological process, cellular component, and molecular function based on *p*-values.

Among the genes affected by the tobacco-flavored e-liquid, the top 20 up- and downregulated genes are listed in Table 5. The upregulated genes included CYP4F3, IL1RL1, PTGS2, and CXCL8, which are related to inflammation; HKDC1, MYEF2 and CYP1A1, which are related to hepatocarcinoma or lung carcinoma; and SLC7A11 and NEFM, which are related to neuronal damage. The downregulated genes included inositol polyphosphate-5-phosphatase D, carbonic anhydrase, proline dehydrogenase, phospholipase, pyrroline-5-carboxylate reductase-like, retinol dehydrogenase 16, and glucokinase. The top 20 genes that were upregulated and downregulated in response to menthol-flavored e-liquids are listed in Table 6. The upregulated genes included IL24, CLU, which are related to apoptosis, and CYP4F3, CCL26, and IL1RL1, which are related to inflammation. The top 20 downregulated genes associated with the menthol-flavored e-liquid included SOX18, which is related to embryonic development; CDH8 and SDK2, which are related to cellular adhesion; and IFITM1, which is related to the immune response.

**Table 5:** List of top 20 up and down regulated transcripts in tobacco-flavored e-liquids.

Gene name	HGNC. ID	Full description of the gene	Fold change (log2)	*p* value
**Up regulated genes**				
CYP4F3	2646	Cytochrome P450 family 4 subfamily F member 3	7.342	0.002
HKDC1	23302	Hexokinase domain containing 1	6.075	0.000
IL1RL1	5998	Interleukin 1 receptor like 1	5.059	0.000
SLC7A11	11059	Solute carrier family 7 member 11	5.024	0.000
AKR1C1	384	Aldo–keto reductase family 1 member C1	4.896	0.000
MCTP1	26183	Multiple C2 and transmembrane domain containing 1	4.889	0.000
KIAA0319	21580	KIAA0319	4.870	0.000
PTGS2	9605	Prostaglandin-endoperoxide synthase 2	4.831	0.000
TRIM36	16280	Tripartite motif containing 36	4.830	0.000
RORA	10258	RAR related orphan receptor A	4.775	0.000
FOSB	3797	FosB proto-oncogene, AP-1 transcription factor subunit	4.734	0.002
KRT34	6452	Keratin 34	4.648	0.001
RRAGD	19903	Ras related GTP binding D	4.603	0.000
NEFM	7734	Neurofilament, medium polypeptide	4.591	0.000
MYEF2	17940	Myelin expression factor 2	4.407	0.000
UNC13A	23150	Unc-13 homolog A	4.402	0.000
RASD1	15828	Ras related dexamethasone induced 1	4.394	0.001
CXCL8	6025	C-X-C motif chemokine ligand 8	4.276	0.000
MAP1B	6838	Microtubule associated protein 1B	4.264	0.000
CYP1A1	2595	Cytochrome P450 family 1 subfamily A member 1	4.173	0.000
**Down regulated genes**				
INPP5D	6079	Inositol polyphosphate-5-phosphatase D	− 6.157	0.000
CA9	1383	Carbonic anhydrase 9	− 5.774	0.000
HPDL	28242	4-hydroxyphenylpyruvate dioxygenase like	− 5.386	0.000
PRODH	9453	Proline dehydrogenase 1	− 4.630	0.000
MMP28	14366	Matrix metallopeptidase 28	− 4.610	0.001
LRFN1	29290	Leucine rich repeat and fibronectin type III domain containing 1	− 4.533	0.000
ZNF488	23535	Zinc finger protein 488	− 4.431	0.000
NKPD1	24739	NTPase, KAP family P-loop domain containing 1	− 4.395	0.000
KANK4	27263	KN motif and ankyrin repeat domains 4	− 4.277	0.000
CDH8	1767	Cadherin 8	− 4.230	0.001
HBQ1	4833	Hemoglobin subunit theta 1	− 4.228	0.000
PLCB2	9055	Phospholipase C beta 2	− 4.159	0.000
KRT81	6458	Keratin 81	− 4.065	0.000
PYCRL	25846	Pyrroline-5-carboxylate reductase-like	− 4.056	0.000
RDH16	29674	Retinol dehydrogenase 16 (all-trans)	− 4.052	0.000
STEAP3	24592	STEAP3 metalloreductase	− 4.030	0.000
ID1	5360	INHIBITOR of DNA binding 1, HLH protein	− 3.948	0.000
GCK	4195	Glucokinase	− 3.933	0.000
KRT7	6445	Keratin 7	− 3.910	0.000
LARGE2	16522	LARGE xylosyl- and glucuronyltransferase 2	− 3.879	0.000

**Table 6:** List of top 20 up and down regulated transcripts in menthol-flavored e-liquids.

Gene name	HGNC. ID	Full description of the gene	Fold change (log2)	*p* value
**Up regulated genes**				
CRYAB	2389	Crystallin alpha B	7.798	0.001
MMP1	7155	Matrix metallopeptidase 1	7.435	0.000
AKR1C1	384	Aldo–keto reductase family 1 member C1	7.430	0.000
HSPA6	5239	Heat shock protein family A (Hsp70) member 6	7.341	0.000
IL24	11346	Interleukin 24	6.707	0.000
HKDC1	23302	Hexokinase domain containing 1	6.501	0.000
ARC	648	Activity regulated cytoskeleton associated protein	6.477	0.000
CYP4F3	2646	Cytochrome P450 family 4 subfamily F member 3	6.443	0.002
HMOX1	5013	Heme oxygenase 1	6.028	0.000
LCP1	6528	Lymphocyte cytosolic protein 1	5.995	0.000
CCL26	10625	C–C motif chemokine ligand 26	5.977	0.001
IL1RL1	5998	Interleukin 1 receptor like 1	5.890	0.000
NEFM	7734	Neurofilament, medium polypeptide	5.699	0.000
SDCBP2	15756	Syndecan binding protein 2	5.233	0.000
RASD1	15828	Ras related dexamethasone induced 1	5.226	0.000
EFR3B	29155	EFR3 homolog B	4.903	0.001
CLU	2095	Clusterin	4.697	0.000
MAP1B	6836	Microtubule associated protein 1B	4.591	0.000
AKR1B10	382	Aldo–keto reductase family 1 member B10	4.563	0.000
SLC7A11	11059	Solute carrier family 7 member 11	4.467	0.000
**Down regulated genes**				
CA9	1383	Carbonic anhydrase 9	− 6.849	0.001
INPP5D	6079	Inositol polyphosphate-5-phosphatase D	− 6.456	0.000
PLCB2	9055	Phospholipase C beta 2	− 4.747	0.000
PADI3	18337	Peptidyl arginine deiminase 3	− 4.680	0.001
MXRA5	7539	Matrix remodeling associated 5	− 4.641	0.001
RDH16	29674	Retinol dehydrogenase 16 (all-trans)	− 4.583	0.001
ALDH3B2	411	Aldehyde dehydrogenase 3 family member B2	− 4.395	0.002
MX2	7533	MX dynamin like GTPase 2	− 4.356	0.000
HAGHL	14177	Hydroxyacylglutathione hydrolase-like	− 4.279	0.000
RARRES3	9869	Retinoic acid receptor responder 3	− 4.273	0.000
MAMDC4	24083	MAM domain containing 4	− 4.257	0.000
CAPS	1487	Calcyphosine	− 4.245	0.000
IFITM1	5412	Interferon induced transmembrane protein 1	− 4.207	0.000
HBQ1	4833	Hemoglobin subunit theta 1	− 4.162	0.000
CDH8	1767	Cadherin 8	− 4.114	0.001
PRODH	9453	Proline dehydrogenase 1	− 4.113	0.000
SDK2	19308	Sidekick cell adhesion molecule 2	− 4.085	0.001
MMP28	14366	Matrix metallopeptidase 28	− 4.084	0.000
CYP24A1	2602	Cytochrome P450 family 24 subfamily A member 1	− 4.082	0.000
SOX18	11194	SRY-box 18	− 4.063	0.000

### Direct signaling pathways among DEGs following e-liquid exposure

**Figure 4:**
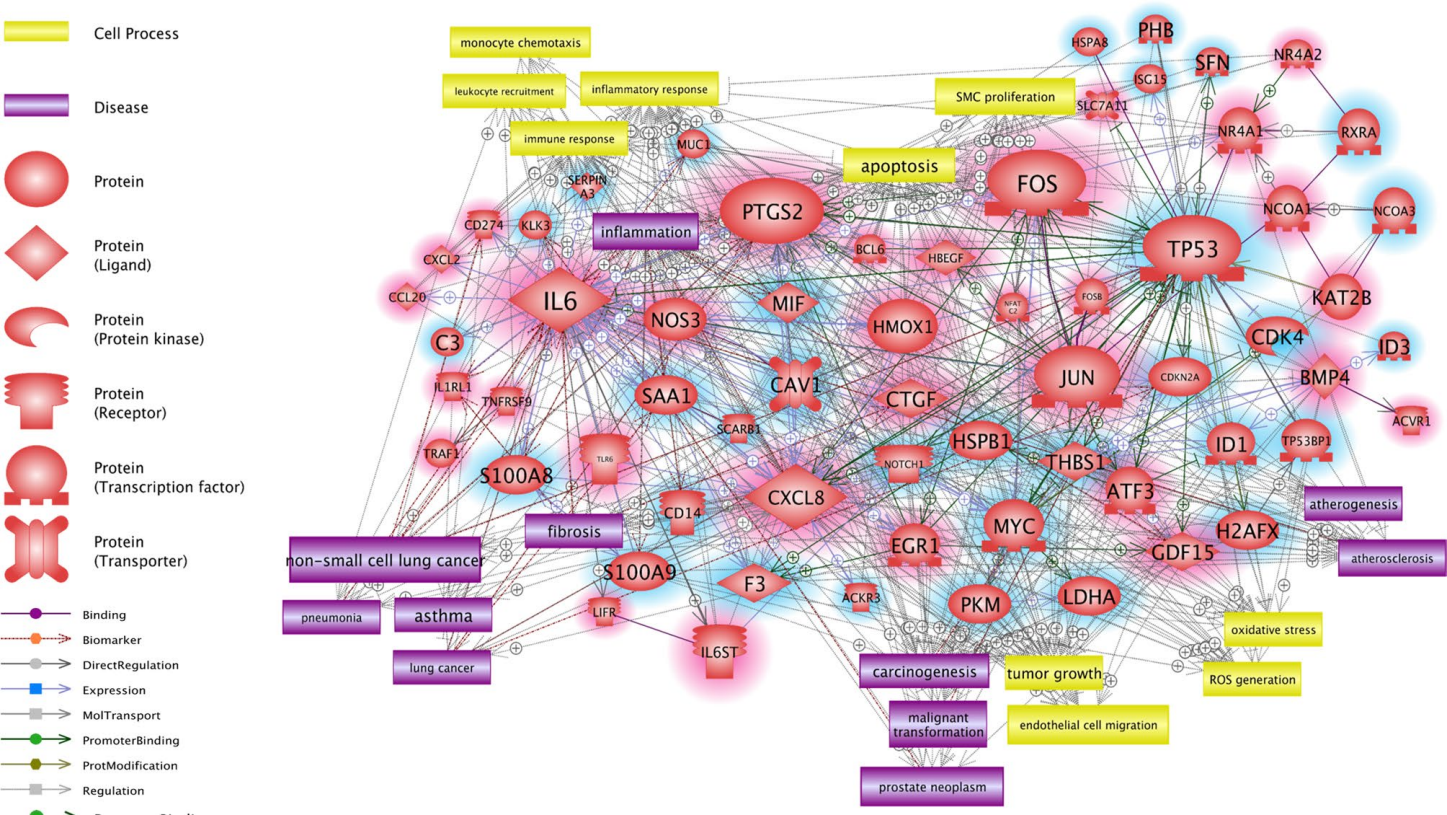
Direct signaling pathways, cell processes and disease-related signaling pathways among the upregulated and downregulated genes identified in response to tobacco-flavored e-liquid. Genes with red shading are upregulated, and genes with blue shading are downregulated. The genes that showed high connectivity in the pathways included IL6 (interleukin 6), PTGS2 (prostaglandin-endoperoxide synthase 2), CXCL8 (C-X-C motif chemokine ligand 8), JUN (Jun proto-oncogene), FOS (Fos proto-oncogene), and TP53 (tumor protein 53). Among these genes, IL6, PTGS2 and CXCL8 are involved in inflammatory pathways, and JUN, FOS, and TP53 are transcription factors associated with numerous interactions. Cell processes and diseases related to inflammation (red rectangle), carcinogenesis (blue rectangle), oxidative stress (orange rectangle), lung disease (brown rectangle) and arterial disease (purple rectangle) showed numerous relationships in the biological signaling networks among the DEGs of the tobacco-flavored e-liquid-treated group. Schematic legends are located on the left side of the figure. Up- and downregulated genes are highlighted with red and blue, respectively.

**Figure 5:**
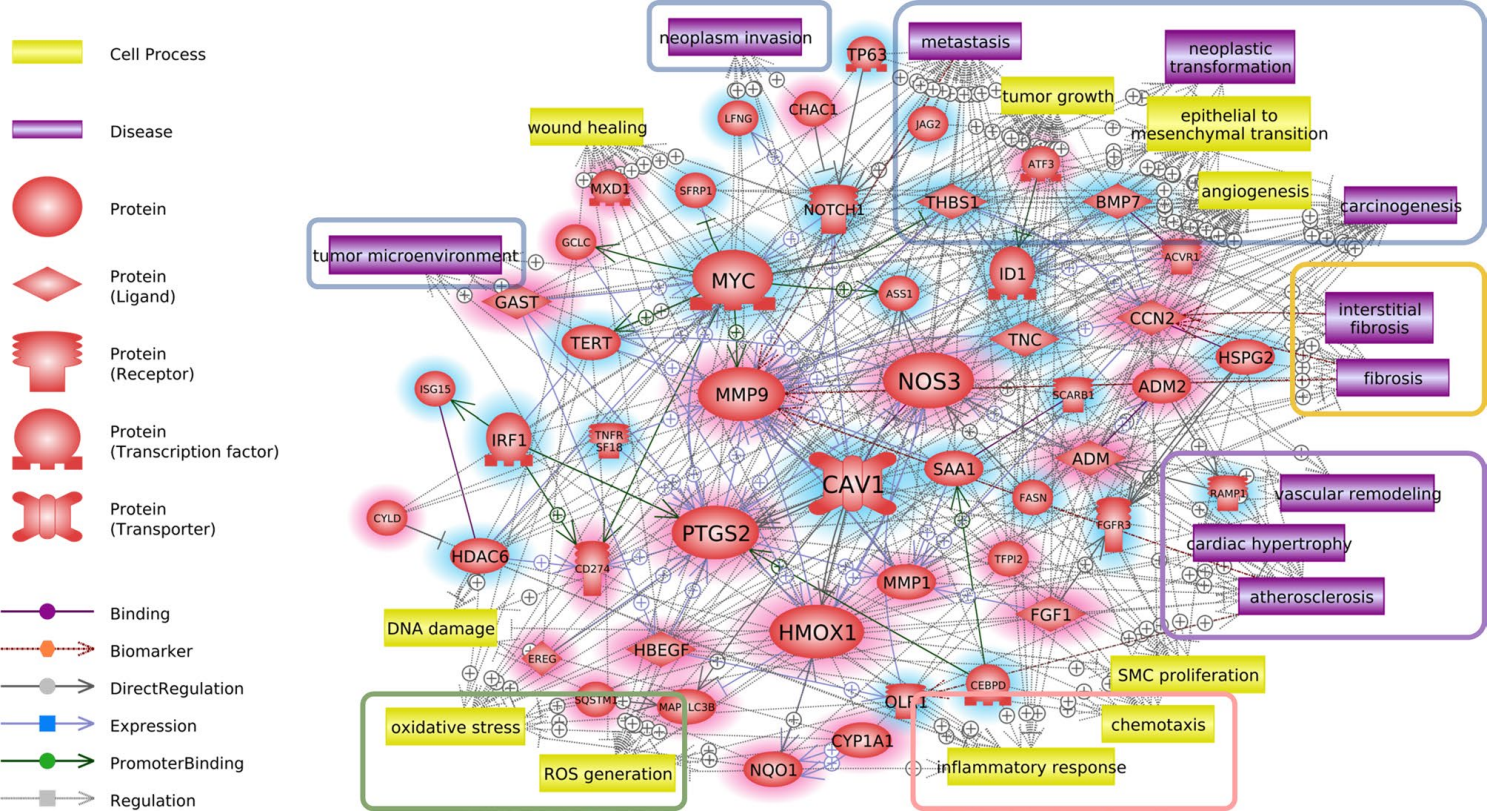
Direct signaling pathways, cell processes and disease-related signaling pathways among the upregulated and downregulated genes identified in response to menthol-flavored e-liquid. Genes with red shading are upregulated, and genes with blue shading are downregulated. MMP9 (matrix metallopeptidase 9), PTGS2 (prostaglandin-endoperoxide synthase 2), MYC (MYC proto-oncogene, bHLH transcription factor), HMOX1 (heme oxygenase 1), NOS3 (nitric oxide synthase 3), and CAV1 (caveolin 1) were predicted as key genes of the direct signaling pathways among the DEGs following menthol-flavored e-liquid exposure. Cell processes and diseases related to cancer (blue rectangle), inflammation (red rectangle), oxidative stress (green rectangle), tissue fibrosis (orange rectangle) and cardiovascular disease (purple rectangle) showed numerous relationships in the biological signaling networks among the DEGs of the menthol-flavored e-liquid-treated group. Schematic legends are located on the left side of the figure. Up- and downregulated genes are highlighted with red and blue, respectively.

The molecular signaling networks among the genes that were differentially expressed in response to the tobacco-flavored e-liquids were analyzed to predict the relevant molecular pathways. The direct interaction pathways among the upregulated and downregulated genes are demonstrated in Fig. 4 and the supplementary information (SI). Various genes were related to each other through several key regulator genes. Among the upregulated genes, IL6, PTGS2, FOS, CXCL8, and JUN showed high connectivity in the pathways; among the downregulated genes, TP53 showed high connectivity in the pathways. The molecular signaling networks among the genes that were differentially expressed in response to the menthol-flavored e-liquid were also analyzed to predict the relevant molecular pathways (Fig. 5 and SI). MMP9 (matrix metallopeptidase 9), PTGS2 (prostaglandin-endoperoxide synthase 2), HMOX1 (heme oxygenase 1), and NOS3 (nitric oxide synthase 3) were upregulated, while MYC (MYC proto-oncogene, bHLH transcription factor) and CAV1 (caveolin 1) were downregulated. These genes were predicted to be key genes of the direct signaling pathways among the DEGs following menthol-flavored e-liquid exposure.

### Cell process- and disease-related biological pathways among genes identified in response to e-liquid exposure

To demonstrate the effect of the tobacco-flavored e-liquid on human middle ear epithelial cells, we analyzed cellular process- and disease-related pathways. We found that the tobacco-flavored e-liquid-related cellular processes included monocyte chemotaxis, leukocyte recruitment, the inflammatory response, the immune response, apoptosis, SMC proliferation, tumor growth, endothelial cell migration, oxidative stress and ROS generation. Several cell processes and diseases related to inflammation (red rectangle), carcinogenesis (blue rectangle), oxidative stress (orange rectangle), lung disease (brown rectangle) and arterial disease (purple rectangle) showed relationships with the biological signaling networks among the DEGs of the tobacco-flavored e-liquid-treated group (Fig. 4). In the menthol-flavored e-liquid-treated group, numerous cell processes and diseases related to cancer (blue rectangle), inflammation (red rectangle), oxidative stress (green rectangle), tissue fibrosis (orange rectangle) and cardiovascular disease (purple rectangle) showed relationships with the biological signaling networks among the DEGs (Fig. 5).

### qRT-PCR expression levels of the potential biomarkers of e-liquid exposure

Based on the RNA-sequence and pathway analyses, we investigated several genes (IL6, PTGS2, FOS, CXCL8, JUN and TP53) as candidate tobacco-flavored e-liquid-responsive biomarkers.

**Figure 6:**
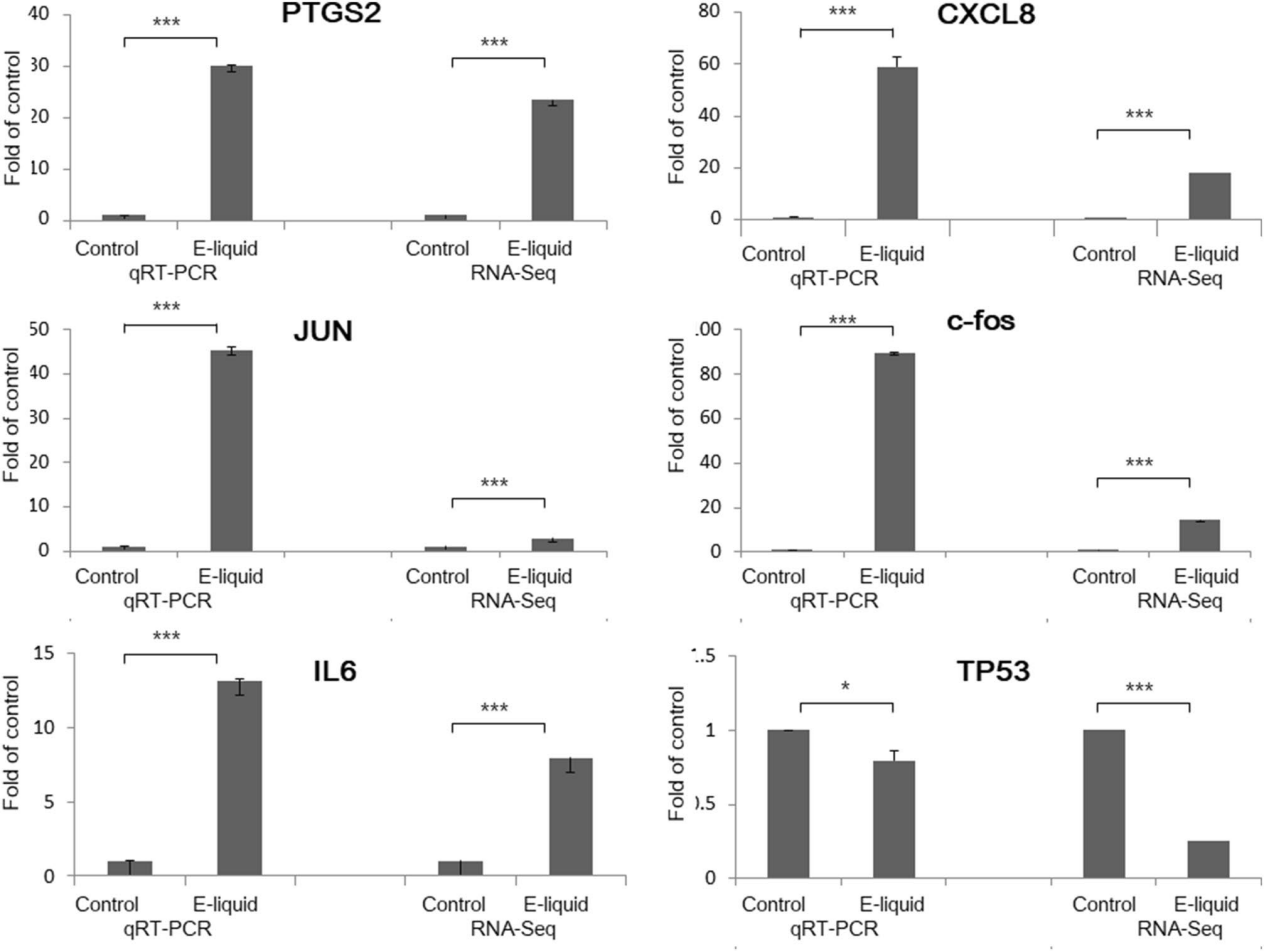
Tobacco-flavored e-liquid-induced genes validated by quantitative real-time polymerase chain reaction (qRT-PCR). In accordance with the RNA sequencing results, the mRNA expression levels of IL6, PTGS2, FOS, CXCL8, and JUN were increased, whereas that of TP53 was decreased.

The selection of key genes in the analyzed pathways was performed based on the local connectivity; Pathway Studio provides the number of relationships with neighbors as local connectivity, which is considered as a parameter for scoring the significance of the entity in the pathway. The top six genes that showed high local connectivity values were selected as the key genes in the pathway. To validate the RNA sequencing results, we examined the expressed transcript levels by qRT-PCR. In accordance with the RNA sequencing results, the expression levels of IL6, PTGS2, FOS, CXCL8, and JUN were found to be increased, whereas that of TP53 was found to be decreased (Fig. 6).

## Discussion

E-cigarettes are evolving and diversifying, and their use among youth and young adults is a major public health concern. While e-cigarette products and their patterns of use are changing quickly, the associated health effects are not completely understood. Since flavoring agents are reported to contribute to the observed cytotoxicity, among other components of e-liquids^18–21^, it would improve the understanding of the effect of e-cigarettes on human organs and bodies to study their transcriptomes at the gene level according to exposure to different flavors. In the current study, we identified the genes and pathways showing alterations in expression upon exposure to e-cigarettes. Furthermore, we investigated the direct interactions between genes and related cellular processes or diseases after exposure to e-cigarettes. Our data demonstrated that e-liquids provoke changes in gene expression in the human middle ear and that there are both similarities and differences in gene expression associated with different flavors.

In the KEGG pathway analysis, both tobacco-flavored and menthol-flavored e-liquid exposure was associated with genes that are related to bladder cancer: CXCL8, HBEGF, CDKN2A, FGFR3, THBS1, CDK4, MYC, TYMP, MMP9, and MMP1. Additionally, genes related to small cell lung cancer, such TRAF1, PTGS2, COL4A2, LAMA5, LAMC3, LAMB3, RXRA, TP53, CDK4, MYC, and PIK3R2, were identified under exposure to both flavors. In the menthol-flavored e-liquid group, genes that are linked with ECM-receptor interactions in small cell lung cancer (COL4A2, LAMB3, LAMA5, and LAMC3) and genes associated with proteoglycans in cancer (CAV1, WNT10B, TFAP4, MMP9, HSPG2, FLNC, ITPR1, CTSL, SMO, ANK1, PLCG2, HBEGF, THBS1, MYC, and PIK3R2) were identified. In the network analysis, diseases such as non-small-cell lung cancer, lung cancer, malignant transformation and prostate neoplasm were associated with exposure to tobacco-flavored e-cigarettes. Moreover, when cells were exposed to menthol-flavored e-liquid, cellular processes related to carcinogenesis was enhanced compared to the results of exposure to the tobacco-flavored e-liquid. Currently, there is no strong evidence or studies suggesting a role of electronic cigarettes in the pathogenesis of cancer^3,22,23^. One in vitro study indicated that electronic cigarette vapors induced double-strand breaks in DNA in head and neck squamous cell carcinoma cell lines that, if unrepaired, would result in chromosomal rearrangement and carcinogenesis^24^. Considering the relatively brief history of these products compared to the prolonged progression of carcinogenesis, it would be premature to discuss the long-term effect of electronic cigarette use. Nevertheless, a transcriptomic analysis can help to identify the relevant effects by revealing changes in gene expression levels.

In the current study, when HMEEC-1 were exposed to tobacco-flavored e-liquid, the top twenty upregulated genes included CYP4F3, IL1RL1, PTGS2, and CXCL8, which are related to inflammation. When cells were treated with menthol-flavored e-liquid, genes associated with inflammation (CYP4F3, CCL26, and IL1RL1) were upregulated, but genes related to apoptosis (IL24, CLU) also showed increases. When we analyzed the molecular signaling network among genes that were differentially expressed in response to both tobacco-flavored and menthol-flavored e-liquids, both groups were found to be enriched in cellular processes such as inflammation, carcinogenesis, and oxidative stress. However, we found that the specific genes involved were different; IL6, PTGS2, FOS, CXCL8, JUN and TP53 were the main key regulator genes in the tobacco-flavored e-liquid group, while MMP9, PTGS2, HMOX1, NOS3, MYC, and CAV1 were the main regulator genes in the menthol-flavored e-liquid group. These findings suggest that e-liquids affect various activities and that different flavors impact numerous pathways.

In the pathway analysis, interstitial fibrosis was a newly predicted pathway among the cellular processes associated with menthol-flavored e-liquid exposure. Additionally, in the KEGG analysis, unlike tobacco-flavored e-liquid exposure, menthol-flavored e-liquid exposure was related to ECM-receptor interactions and focal adhesions. Cell proliferation is subject to many levels of control, but it is becoming apparent that mechanical signaling through the cytoskeleton link between focal adhesions and regulators of cellular contractility contributes to the regulation of cell proliferation^25,26^. These findings might explain the difference in cell viability between tobacco- versus menthol-flavored e-liquid exposure.

E-cigarettes might be less cytotoxic than conventional cigarettes^27–30^; however, we cannot overlook the unexpected health effects resulting from e-cigarette exposure. Other researchers have reported incompatible findings regarding the significant effects of e-cigarettes found in transcriptomic analysis. In studies with HBE cells (BEAS-2B), some authors have reported that e-cigarette vapor (e-vapor) exposure results in lower toxicity than conventional cigarette smoking and that no significant differences in gene expression could be found^27^. Another study reported that e-vapors are not benign and that they elicit discrete transcriptomic signatures in HBE cells, such as alterations in phospholipid and fatty acid triacylglycerol metabolism pathways, cytochrome P450 function, retinoid metabolism, and nicotine catabolism^17^. In studies conducted in mouse heart tissue and a human gingival epithelial organotypic culture system with Tobacco Heating System 2.2 (THS2.2), which heats tobacco instead of burning it and generates an aerosol similar to that of an e-cigarette, the authors reported no changes or fewer changes in gene expression compared to conventional cigarette exposure^17,30^. However, these studies adopted different doses of e-cigarette exposure, analysis time points and delivery methods, and they mostly compared their data with the results of conventional cigarette exposure.

In the present study, both tobacco-flavored and menthol-flavored e-liquids affected KEGG pathways involved in glucose, amino acid and lipid metabolism, which could be related to the regulation of many physiological processes. Additionally, the solvent (PG, VG) itself showed cytotoxicity in the CCK analysis cell viability test and induced differential gene expression compared to the control according to the heatmap and the volcano plot. Another report showed that the e-cigarette exposure altered the expression of rhythmic genes in a way that could be translated into systemic biological alterations and that the major solvents used in e-cigarettes (PG, VG) had unsuspected effects on gene expression related to the molecular clock^31^.

Our experiment was performed in the HMEEC line to identify the effect of e-cigarettes on gene expression, related cellular processes and gene signaling pathways. One limitation of this study is that we did not identify the effect of the vaporized e-liquid, so the data may not reflect the changes that occur in human tissues. However, the results of the present study will help future studies examine the mechanisms underlying the effects of e-cigarettes on the human middle ear and upper airway.

## Methods

### Preparation of e-liquids

We analyzed 73 bottles of e-liquids from 12 different brands purchased from local retailers after reviewing various rapidly changing available products^5^. We selected the two most popular and most reliable (according to previous experiments) e-liquids, including one tobacco-flavored e-liquid and one menthol-flavored e-liquid. The bottles containing the liquids were kept at room temperature and protected from light until they were used for analysis.

### HMEEC culture

An HMEEC-1 cell line (kindly provided by Dr. David J. Lim, House Ear Institutes, Los Angeles, CA, USA) immortalized with the E6/E7 genes of human papilloma virus type 16 was maintained in a mixture of Dulbecco's modified Eagle's medium (Invitrogen, Carlsbad, CA, USA), bronchial epithelial basal medium (BEBM) (Lonza, Walkersville, MD, USA) (1:1) and other growth medium supplements, including bovine pituitary extract (52 μg/mL), hydrocortisone (0.5 μg/mL), human epidermal growth factor (hEGF; 0.5 ng/mL), epinephrine (0.5 mg/mL), transferrin (10 μg/mL), insulin (5 μg/mL), triiodothyronine (6.5 ng/mL), retinoic acid (0.1 ng/mL), gentamycin (50 μg/mL), and amphotericin B (50 ng/mL)^32^.

To study the effects of the e-liquids, the cells were grown to 60% confluence in 96-well culture plates (SPL Life Science, Korea) at 37 °C in a carbon dioxide-enriched (95% air, 5% CO_2_) humidified atmosphere. The HMEEC-1 cells were plated in 96-well plates (1 × 10^4^ cells/well) for cell viability assays or 6-well plates (5 × 10^5^ cells/well) for RNA-seq analysis. The next day, when the cells reached 80–90% confluence, the cells were starved with serum-free medium for 2 h, then exposed to the e-liquids and subsequently incubated for 24 h. The control groups were not exposed to the e-liquids or PG/VG treatment.

### Cell viability after exposure to various components of e-liquids

Cell viability was measured using a cell counting kit-8 (CCK-8, Dojindo Laboratories, Kumamoto, Japan). HMEEC-1 cells were seeded in 96-well plates, with 1 × 10^4^ cells in each well. The following day, the cells were treated with the tobacco-flavored e-liquid or the menthol-flavored e-liquid at 0, 0.01, 0.1, 1, 2, 3, 4, or 5%; the solvent (PG:VG = 5:5) at 0, 0.01, 0.1, 1, 2, 3, 4, or 5%; or nicotine at 0, 0.01, 0.005, 0.01, 0.5, or 0.1%. After 24 h, the CCK-8 solution was added to each well, and the plates were incubated for 150 min at 37 °C. The optical density was measured at 450 nm using a microplate reader (Spectra Max plus 384; Molecular devices, Sunnyvale, CA, USA). ED50plus v 1.0 software was used to calculate the IC50 (half maximal inhibitory concentration).

### RNA sequencing

HMEEC-1 cells (5 × 10^5^) were cultured in 6-well plates (SPL Life Science, Korea) and grown for 24 h. When the cells reached 80% confluence, they were starved for 2 h and exposed to the e-liquids at the IC_50_ concentration (4.50% solvent (PG:VG = 5:5), 0.07 mg/mL nicotine, 3.02% tobacco-flavored e-liquid and 1.62% menthol-flavored e-liquid) for 24 h. The nontreated group was used as a control. Two replicates of five samples were obtained (Control-1st, Control-2nd, Menthol-1st, Menthol-2nd, Nicotine-1st, Nicotine-2nd, PV-1st, PV-2nd, Tobacco-1st, and Tobacco-2nd) to extract total RNA from tissue samples for further data analysis.

Total RNA was isolated from the tissue samples using the phenol-based (TRIzol) method. One microgram of total RNA was processed to prepare the mRNA sequencing library using a TruSeq stranded mRNA sample preparation kit (Illumina, San Diego, CA) according to the manufacturer’s instructions. The products were then purified and enriched via PCR to generate the final cDNA library. Finally, the quality and band size of the libraries were assessed using an Agilent 2100 Bioanalyzer (Agilent). The libraries were quantified by qPCR using the CFX96 Real-Time System (Bio-Rad). After normalization, sequencing of the prepared libraries was conducted on the NextSeq system 500 (Illumina) with 75 bp paired-end reads.

### Transcriptome data analysis

Potential sequencing adapters within the raw reads were trimmed by Skewer ver 0.2.2^33^. The cleaned high-quality reads after the trimming of sequencing adapters were mapped to the human reference genome (EnsEMBL, GRC37) using STAR (ver 2.5)^34^. Since the sequencing libraries were prepared in a strand-specific manner by using Illumina’s strand-specific library preparation kit, the strand-specific library option –library-type = fr-firststrand was applied in the mapping process.

To quantify the mapped reads against the reference genome to obtain gene expression values, Cuffquant (Cufflinks ver 2.2.1) was used with the strand-specific library option –library-type = fr-firststrand and other default options. The gene annotation of the reference genome mm10 from the UCSC Genome Browser (https://genome.ucsc.edu) in GTF format was used for a gene model assignment, and the expression values were calculated in fragments per kilobase of transcript per million fragments mapped (FPKM) units. The differentially expressed genes (DEGs) between the two selected biological conditions were analyzed with Cuffdiff software in the Cufflinks package with the strand-specific library option-library-type = fr-firststrand and remaining default options (Control-VS-Menthol, Control-VS-Nicotine, Control-VS-PV, Control-VS-Tobacco, Menthol-VS-Nicotine, Menthol-VS-PV, Menthol-VS-Tobacco, Nicotine-VS-PV, Nicotine-VS-Tobacco, PV-VS-Tobacco). To compare the expression profiles among the samples, the normalized expression values of selected DEGs were clustered in an unsupervised manner with in-house R scripts. DEGs were identified according to a significance threshold of a q-value (false discovery rate) < 0.05. To identify the biological functional roles of the differences in gene expression between the compared biological conditions, a gene set overlap test between the analyzed DEGs and the functionally categorized genes (according to GO biological processes, KEGG pathways, and transcription factor binding target gene sets) was performed using the DAVID tool^35^. The R program was used for the hierarchical clustering of different samples, heatmap of clustering, and the visualization of various types of plots of the gene expression profile^36^.

### Pathway analysis

The molecular pathways of the DEGs identified by mRNA sequencing were analyzed using Pathway Studio version 12.0 (Elsevier, USA). Based on a text-mining algorithm and the scientific literature, this web-based software provides a schematic output consisting of the biological relationships among the imported gene lists as well as an expanded analysis of relevant cell processes and diseases (PMID: 14594725). All relationships among genes, cell processes and diseases were trimmed based on the number of references (> 10) and the reliability of the reference sentences. The selection of key genes in the analyzed pathways was performed based on the local connectivity; Pathway Studio provides the number of relationships with neighbors as local connectivity, which is considered as a parameter for scoring the significance of the entity in the pathway. The top six genes that showed high local connectivity values were selected as the key genes in the pathway.

### Quantitative real-time polymerase chain reaction (qRT-PCR)

To validate gene expression levels, qRT-PCR was performed on six genes that showed markedly different expression levels in e-liquid-treated HMEECs according to RNA sequencing analysis: prostaglandin-endoperoxide synthase 2 [PTGS2], C-X-C Motif Chemokine Ligand 8 [CXCL8], Jun Proto-Oncogene [JUN], Fos proto-oncogene [c-fos], interleukin 6 [IL6] and tumor protein p53 [TP53]). The primers designed for these assays are shown in SI.

Total RNA was extracted from each cell group using the RNeasy Mini Kit (Qiagen, Hilden, Germany), and DNase I (Qiagen) was used to remove genomic DNA from the RNA samples during RNA preparation according to the manufacturer’s instructions. cDNA was synthesized with Superscript II reverse transcriptase (Bioneer, Inc., Daejeon, Korea). RNA yield and purity were determined with a Nanodrop ND-2000 spectrophotometer (Thermo Fisher Scientific, Waltham, USA). The quantitation of mRNA expression was carried out using the ABI Prism 7300 real-time polymerase chain reaction (PCR) system (Applied Biosystems, Foster, CA, USA). PCR amplification was performed in a 20-µL final reaction mixture containing 1 µL of 5 pmol forward and reverse primers, 1 µL of cDNA, and Power SYBR™ Green PCR Master Mix (Life Technologies, Carlsbad, CA, USA). The PCR mixtures were incubated at 95 °C for 15 s and 60 °C for 1 min, followed by amplification for 45 cycles. The target mRNA expression levels were normalized to those of the gene encoding endogenous glyceraldehyde-3-phosphate dehydrogenase (*GAPDH*), and relative gene expression in the experimental groups was calculated via the 2^(−∆∆Ct)^ method.

### Statistical analysis

All values are represented as the mean ± SD. For data analysis, we used the SPSS 24.0 statistical program. The Kruskal–Wallis test was employed for comparisons between two groups, and ANOVA was used to compare multiple groups in the cell viability assay. A *p* value of < 0.05 was considered statistically significant. For multiple comparisons, Bonferroni correction was performed.

## Supplementary information

Supplementary information.

Supplementary information 2.

Supplementary information 3.
