# Supplementary material for: Transcriptomic analysis of tobacco-flavored E-cigarette and menthol-flavored E-cigarette exposure in the human middle ear
Source: Sci Rep. 2020 Nov 27;10:20799. doi: 10.1038/s41598-020-77816-2 (PMC7699635; doi:10.1038/s41598-020-77816-2)
Supplement: Supplementary file 1 — Supplementary information. [file 41598_2020_77816_MOESM1_ESM.docx]

**Transcriptomic analysis of Tobacco-flavored E-cigarette and Menthol-flavored E-cigarette Exposure in the Human Middle Ear**

Jae-Jun Song^1^, Yoon Young Go^1^, Jong kyou Lee ^2^, Bum sang Lee^2^, Su-kyoung Park ^2^, Harry Jung^3^, Jun Ho Lee^2^, Jiwon Chang^2*^

^1^Department of Otolaryngology-Head and Neck Surgery, Korea University College of Medicine, Seoul, Korea

^2^Department of Otolaryngology-Head and Neck Surgery, Hallym University College of Medicine, Seoul, Korea

^3^Institute of New Frontier Research Team, Hallym Clinical and Translation Science Institute, Hallym University, Chuncheon, Republic of Korea

Jae-Jun

*Corresponding author:

Jiwon Chang, MD, PhD

Department of Otolaryngology-Head and Neck Surgery,

Kangnam Sacred Heart Hospital, Hallym University College of Medicine

948-1, Daerim 1-dong, Yeongdeunpo-gu, Seoul, Korea, 150-950

Tel. (82)-2-829-5217, Fax (82)-2-842-5217

E-mail: brune77@naver.com

**Supplementary materials**

**
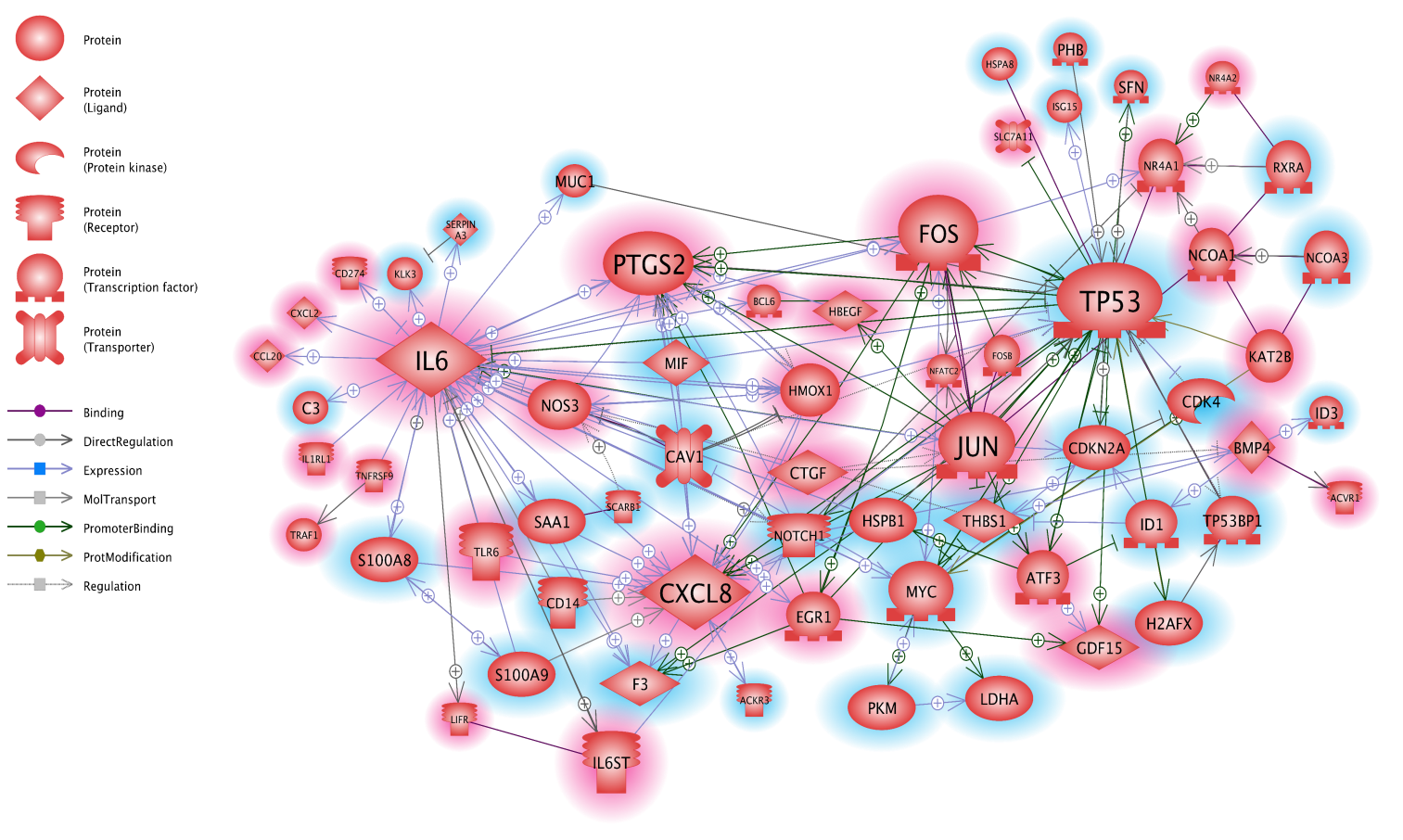
**

**Supplementary Figure 1. Direct signaling pathway among DEGs following tobacco-flavored e-liquid exposure.** IL6 (interleukin 6), PTGS2 (prostaglandin-endoperoxide synthase 2), CXCL8 (C-X-C motif chemokine ligand 8), JUN (Jun proto-oncogene), FOS (Fos proto-oncogene), and TP53 (tumor protein 53) were predicted as key genes of the direct signaling pathway among DEGs following tobacco-flavored e-liquid exposure. Schematic legends are located in left side of the figure. Up- and down-regulated genes were highlighted with red and blue, respectively. Schematic legends are located in left side of the figure.


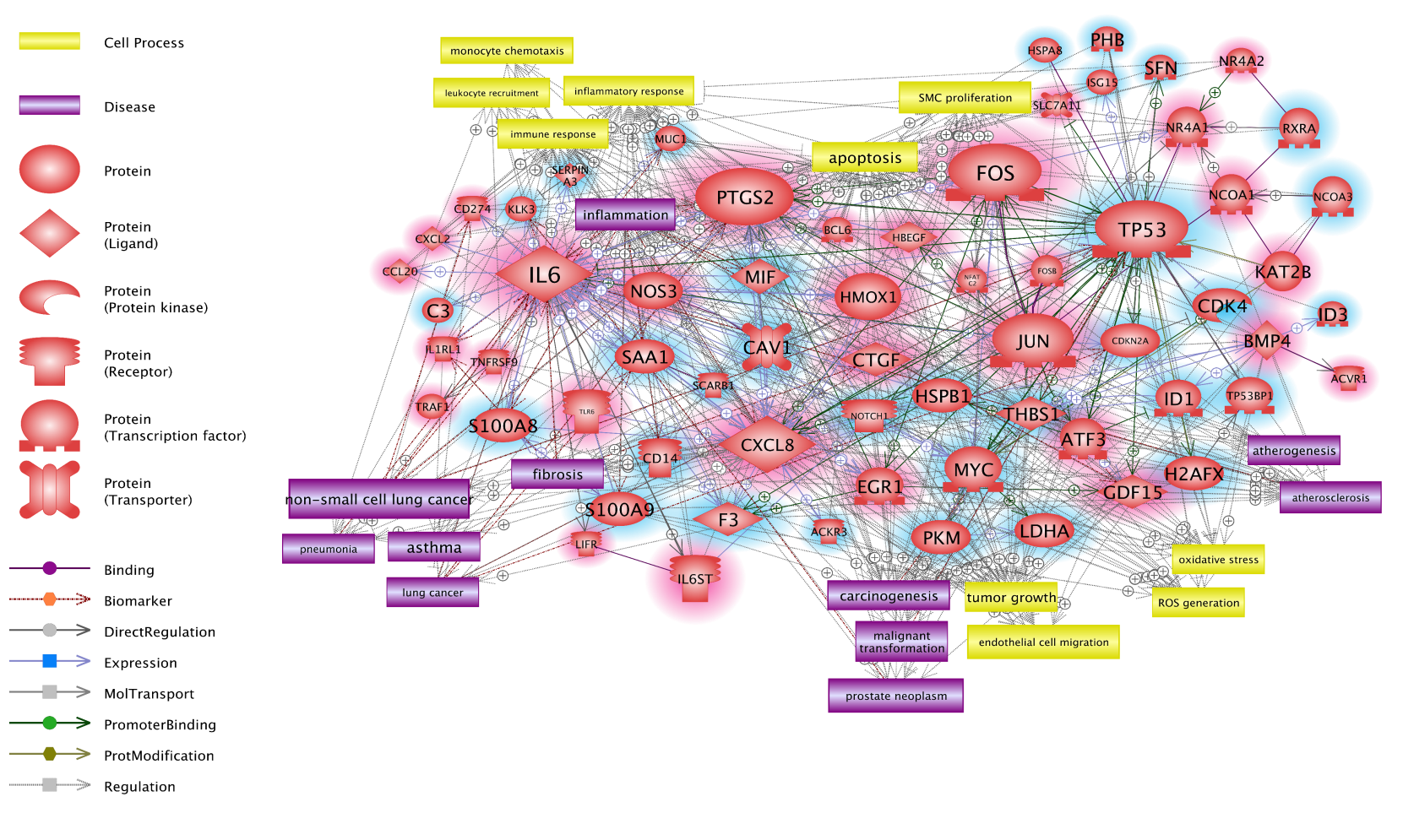


**Supplementary Figure 2. Cell processes and diseases-related biological pathways among genes in response to tobacco-flavored e-liquid.** Cell processes and diseases relevant with inflammation (red-colored rectangle), carcinogenesis (blue-colored rectangle), oxidative stress (orange-colored rectangle), lung disease (brown-colored rectangle) and arterial disease (purple-colored rectangle) showed high number of relation in the biological signaling networks among the DEGs of tobacco-flavored e-liquid-treated group related. Schematic legends are located in left side of the figure. Up- and down-regulated genes were highlighted with red and blue, respectively.

**
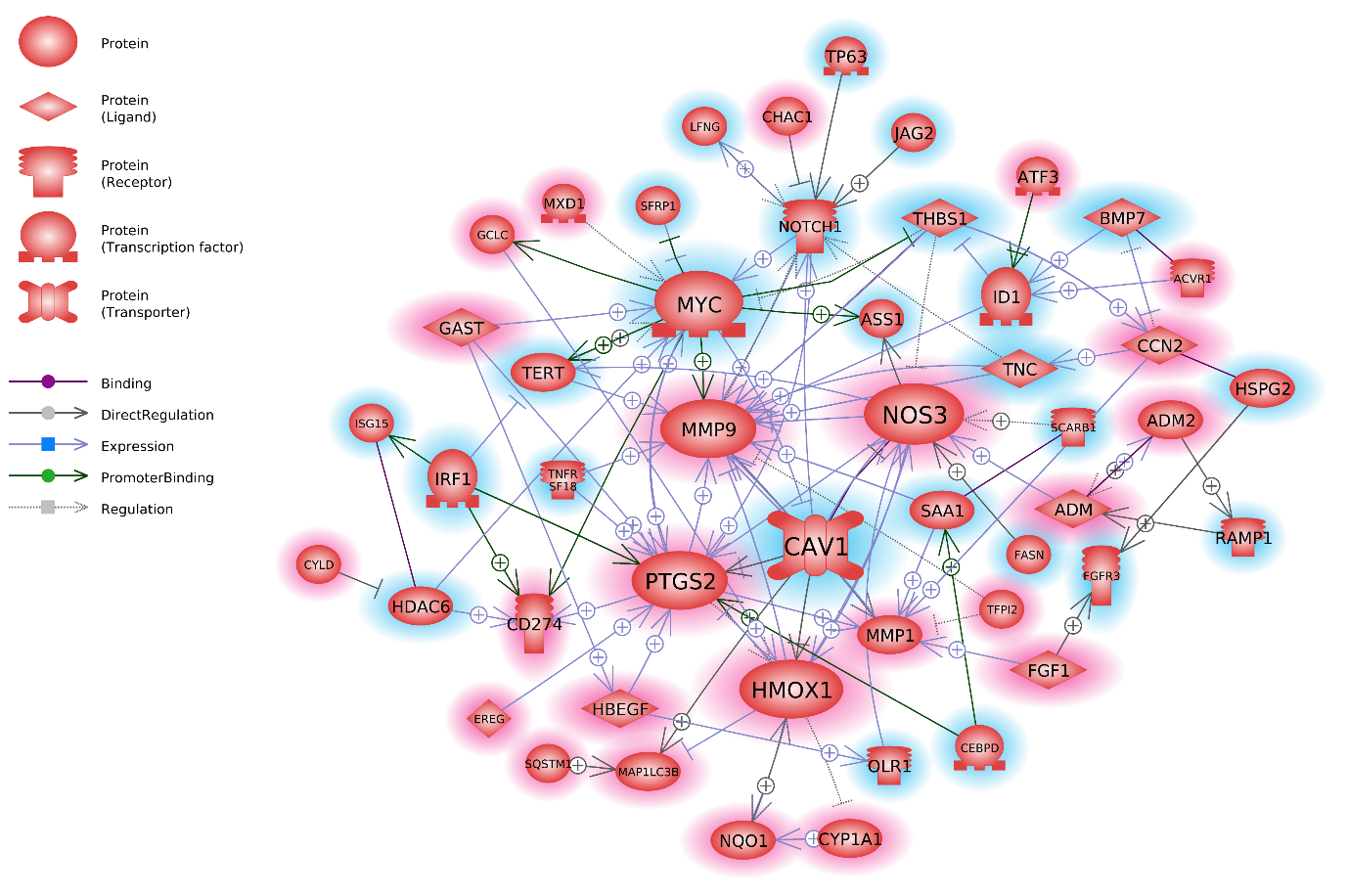
**

**Supplementary Figure 3. Direct signaling pathway among DEGs following menthol-flavored e-liquid exposure.** MMP9 (matrix metallopeptidase 9), PTGS2 (prostaglandin-endoperoxide synthase 2), MYC (MYC proto-oncogene, bHLH transcription factor), HMOX1 (heme oxygenase 1), NOS3 (nitric oxide synthase 3), and CAV1 (caveolin 1) were predicted as key genes of the direct signaling pathway among DEGs following menthol-flavored e-liquid exposure. Schematic legends are located in left side of the figure. Up- and down-regulated genes were highlighted with red and blue, respectively. Schematic legends are located in left side of the figure.


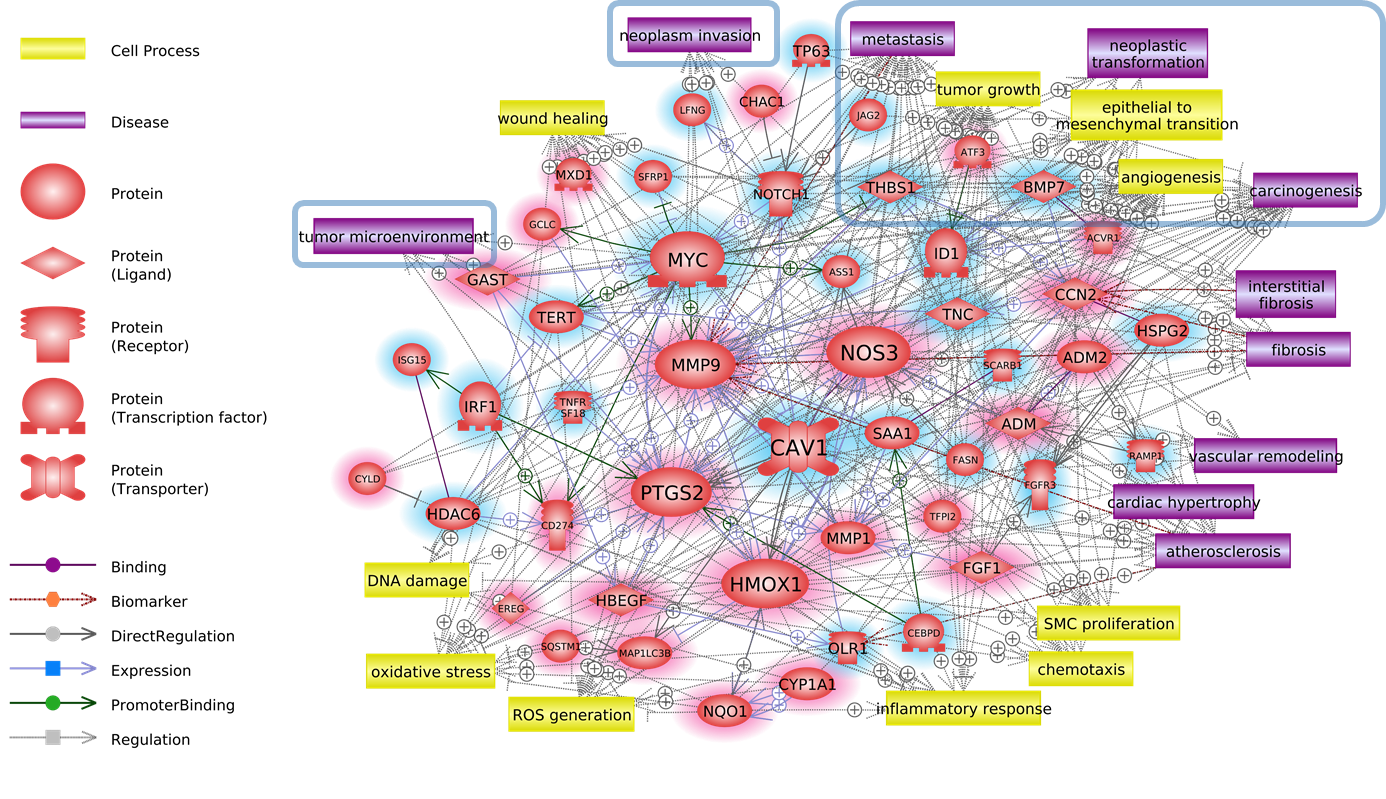
**Supplementary Figure 4. Cell processes and diseases-related biological pathways among genes in response to menthol-flavored e-liquid.** Cell processes and diseases relevant with cancer (blue-colored rectangle) showed high number of relation in the biological signaling networks among the DEGs of menthol-flavored e-liquid-treated group related. Schematic legends are located in left side of the figure. Up- and down-regulated genes were highlighted with red and blue, respectively.

Supplementary Table 1. Sequences of primer oligonucleotides used for quantitative real-time PCR

| No. | Species | Oligomer | Site | Sequence |
| --- | --- | --- | --- | --- |
| 1 | human | TP53 | Forward | 5'- AGA CCT GTG GGA AGC GAA AA - 3' |
|  |  |  | Reverse | 5'- TCA TCC ATT GCT TGG GAC GG -3' |
| 2 | human | PTGS2 | Forward | 5' -ATG CTG ACT ATG GCT ACA AAA GC -3' |
|  |  |  | Reverse | 5'- TCG GGC AAT CAT CAG GCA C -3' |
| 3 | human | CXCL8 | Forward | 5'- ACT GAG AGT GAT TGA GAG TGG AC -3' |
|  |  |  | Reverse | 5'- ACA ACC CTC TGC ACC CAG TT -3' |
| 4 | human | JUN | Forward | 5'- TCG ACA TGG AGT CCC AGG A - 3' |
|  |  |  | Reverse | 5'- GGC GAT TCT CTCC CAG CTT CC - 3' |
| 5 | human | c-foS | Forward | 5'- TCA CCC GCA GAC TCC TTC TC -3' |
|  |  |  | Reverse | 5'- GTG GGA ATG AAG TTG GCA CTG -3' |
| 6 | human | IL-6 | Forward | 5'- TCT TCA GAA CGA ATT GAC AAA CAA A -3' |
|  |  |  | Reverse | 5'- GCT GCT TTC ACA CAT GTT ACT CTT G -3' |
| 7 | human | GAPDH | Forward | 5'-TCG CCC CAC TTG ATT TTG G-3' |
|  |  |  | Reverse | 5'-GCA AAT TCC ATG GCA CCG T-3' |
